# The Inversion Effect for Chinese Characters is Modulated by Radical Organization

**DOI:** 10.1007/s10936-017-9484-0

**Published:** 2017-03-27

**Authors:** Canhuang Luo, Wei Chen, Ye Zhang

**Affiliations:** 1 Institutes of Psychological Sciences, Hangzhou Normal University, Hangzhou, Zhejiang China; 2 Center for Cognition and Brain Disorders, Hangzhou Normal University, No.2318, Yuhangtang Rd, Cangqian, Yuhang District, Hangzhou, 311121 Zhejiang China; 3 Zhejiang Key Laboratory for Research in Assessment of Cognitive Impairments, Hangzhou, Zhejiang China; 4 Science Division, New York University Abu Dhabi, Abu Dhabi, United Arab Emirates

**Keywords:** Chinese character, Character structure, Inversion effect, Configural processing

## Abstract

In studies of visual object recognition, strong inversion effects accompany the acquisition of expertise and imply the involvement of configural processing. Chinese literacy results in sensitivity to the orthography of Chinese characters. While there is some evidence that this orthographic sensitivity results in an inversion effect, and thus involves configural processing, that processing might depend on exact orthographic properties. Chinese character recognition is believed to involve a hierarchical process, involving at least two lower levels of representation: strokes and radicals. Radicals are grouped into characters according to certain types of structure, i.e. left–right structure, top–bottom structure, or simple characters with only one radical by itself. These types of radical structures vary in both familiarity, and in hierarchical level (compound versus simple characters). In this study, we investigate whether the hierarchical-level or familiarity of radical-structure has an impact on the magnitude of the inversion effect. Participants were asked to do a matching task on pairs of either upright or inverted characters with all the types of structure. Inversion effects were measured based on both reaction time and response sensitivity. While an inversion effect was observed in all 3 conditions, the magnitude of the inversion effect varied with radical structure, being significantly larger for the most familiar type of structure: characters consisting of 2 radicals organized from left to right. These findings indicate that character recognition involves extraction of configural structure as well as radical processing which play different roles in the processing of compound characters and simple characters.

## Introduction

The recognition of faces is both less accurate and slower when viewed upside-down. This well-known phenomenon is referred to as the face inversion effect and has been taken as a marker of the configural processing of faces. Configural processing may develop from years of experience with the discrimination of faces in their normal, upright orientation (Maurer et al. 2002). The configural processing of these upright faces could include both the processing of the relation that specifies position properties of facial features (e.g. the eyes are above the nose and the mouth is below the nose) and the processing of the relation that specifies distances between facial features (Tanaka and Sengco 1997; for a review see Maurer et al. 2002). Besides, configural processing may also involve integrating facial features together into a whole (Maurer et al. 2002; McKone 2004). When faces are inverted, perceptual experience for upright faces fails to generalize to unfamiliar orientations, resulting in an inability to extract configural information of faces. It is suggested that face inversion may interfere with different levels of configural processing (Maurer et al. 2002) and result in processing that is solely based on facial features (Peterson and Rhodes 2003; Sagiv and Bentin 2001). However, it remains unknown whether configural processing is as important for recognizing Chinese characters as it is for recognizing faces and to what extent configural processing is disrupted by inversion for character. Recent studies report inversion effects for Chinese characters (Kao et al. 2010; Wang et al. 2011), which suggests an involvement of configural processing in character recognition.

Chinese characters are indeed comparable with faces. They are roughly treated as two-dimensional spatial or pictographic visual patterns (Zhang et al. 2009). The compositional relationship of components in a character is similar to facial features and their inter-relations in faces. Besides, recognizing characters comes to be orientation-specific in skilled readers because of lifetime exposure to the upright orientation, just as the development of perceptual expertise with faces. This predicts that configural processing could be also important for character recognition and would be disrupted for well-learnt characters that are presented in an unfamiliar orientation. Thus, a face-like inversion effect should be observed for Chinese characters, as already reported by both Kao et al. (2010) and Wang et al. (2011). The present study aims to replicate this finding. However, our main purpose is to determine whether the magnitude of the character inversion effect is dependent on what we call ‘radical structure’. Although Chinese characters have similar pictographic and compositional properties with faces, Chinese characters as linguistic scripts are more complex than faces. Firstly, the perceptual organization of Chinese characters are constructed maximally with three levels: stroke, radicals, and structure (Yeh et al. 2003). A number of individual strokes not only compose different characters but also form various types of radicals. Stroke patterns or one single radical can be simple characters in their own right with independent meanings and pronunciations, which are referred to as simple characters. More than two distinct radicals form different compound characters. Secondly, strokes and radicals are arranged in various locations within characters, and the precise arrangement of strokes and radicals has to conform to certain implicit principles of spatial-position regularity, i.e. orthography (Suk-Han Ho et al. 2004; Yeh and Li 2002; Wang et al. 2003). Thus, there is difference in the variation of feature position relations between characters and faces. For faces, the locations of facial features are the same across individual faces(i.e. two eyes are always above a nose). For characters, different radicals that are arranged at various positions have several structures such as horizontal, vertical, open and enclosed structure within compound characters (Yeh et al. 2003, 1997). For example, two radicals can be arranged horizontally (e.g. left–right structure, ), or arranged vertically (e.g. top–bottom structure, ). Simple characters with strokes arranged at different positions have a simple structure, consisting of a single unit or ‘radical’.

Given that Chinese characters vary in the organization of their primary features, we propose that the configural processing of Chinese characters might be dependent on precise orthography; orthography of Chinese characters that we have learnt implicitly for years make our visual system group radicals together and process characters configurally. One explanation of configural processing emphasizes the putative hierarchical nature of internal character representations (Wang et al. 2011; Kao et al. 2010): characters are first parsed into radicals and this level of configuration is calculated from positional relationships between radicals; then radicals are decomposed into strokes and this level of configuration relies on the metric information of stroke arrangements (Wang et al. 2011; Taft et al. 1999). Due to potential differences in the number of hierarchical levels required for the representation of simple and compound characters, the recognition of these two kinds of characters may demand different degrees of configural processing. This leads us to predict that the inversion effects should be greater for compound characters because, while inversion of the simple character disrupts relational information from strokes, inversion of the compound character disrupts the relational information of both radicals and strokes.

An additional, perhaps complementary description of configural processing suggests that a character is perceived as a globally structured pattern. Yeh and her colleagues suggest that the expert recognition of characters requires a process of binding radicals into a single perceptual unit in order to reduce processing load (Yeh et al. 2003; Chen and Yeh 2015). Overall ability to bind radicals into single units is acquired through long-term implicit learning. However, familiarity can vary with type of radical structure and it has been shown that expertise with Chinese character recognition may, for example, facilitate the extraction of the largest unit available; i.e. radicals over strokes and full characters over individual radicals (Yeh 2000; Yeh et al. 2003). However, what happens to configural processing when radical structure varies but their highest hierarchical level remains constant? We suggest that, if expertise is specific to the exact type of radical structure, configural processing should suffer the most for inverted structures that are most familiar. In this study, we take advantage of the fact that Chinese characters of different structures indeed have different degrees of familiarity. Left–right characters are maximally familiar to primary school and university students (Hue 2003; Lui et al. 2010). Of over 6700 daily used characters, around 63% are left–right characters, in contrast to about 23% for top–bottom characters (Guo 1999); while simple characters comprise at most 14% of the total. We predict that, compared to top–bottom and simple characters, inversion in left–right characters may lead to greater disruption of configural structure.

In the present study, we aim to examine whether the hierarchical level or the familiarity of radical organization plays a critical role in Chinese character recognition by using the inversion effect to index the involvement of configural processing. We compare the inversion effect of three types of character structures (simple, left–right, and top–bottom), hypothesizing that: (1) based on the idea that configural processing cumulates across levels of hierarchical representations or relational configuration, a greater inversion effect will be observed in compound relative to simple characters. This will be tested by comparing the inversion effect of simple and top–bottom characters, both of which have similar familiarity with structure; (2) based on the radical-structure explanation, due to higher familiarity with left–right characters, left–right characters will show a stronger inversion effect compared to other types of characters. This will be tested by comparing the inversion effect of left–right and top–bottom characters, since these two types of characters have similar structure complexity in terms of number of radicals. Up to now, few studies have examined the difference in perception between simple and compound characters. As far as we know, Wang et al. (2011) is the only previous study to do so. However, Wang et al.’s study (2011) failed to find an interaction between character structure (simple vs. compound) and orientation (upright vs. inverted) in behavioral performance, neither in reaction time nor accuracy. They failed to consider the potential impact of radical-structure familiarity and did not distinguish left–right from top–bottom structure in the compounds. We suspect that familiarity with structure could have been a confounder in Wang et al.’s study.

In addition, the present study manipulates presentation eccentricity of characters (fovea and parafovea). It is suggested that fluent reading requires both foveal and parafoveal processing (e.g. Schotter et al. 2012; Vasilev and Angele 2016). Parts of information such as the lexicon can be obtained from a parafoveal word with slow extraction speed (Schotter et al. 2012). But what information is critical to process characters in the parafovea is not very clear. Based on the previous studies of parafoveal reading, we speculate that configural processing of printed words may remain in the parafovea. If this speculation is true, processing of inverted characters in the parafovea would suffer most because parafoveal reading may heavily depend on configural information. Thus, we expected that the inversion effect is more obvious in the parafovea than that in the fovea.

## Method

### Participants

Twenty healthy students (5 males) aged 18–20 years (mean age 19 years) were enrolled in the study and paid for their participation. All participants were native Chinese readers. In addition, all participants were right-handed and had normal or corrected-to-normal vision. The experimental procedure performed in the current study was in accordance with the Institutional Review Board of Hangzhou Normal University and with the 1964 Helsinki declaration and its later amendments or comparable ethical standards. Informed consent was obtained from each participant in accordance with the guidelines and approval of the Center for Cognition and Brain Disorders of Hangzhou Normal University.

### Stimuli and Apparatus

All stimuli for this study were selected from the SUBTLEX-CH corpus (Cai and Brysbaert 2010). There were 75 Chinese characters (see Appendix), with 25 characters for each type of the 3 structures, which are left–right characters (LRC), top–bottom characters (TBC), and simple characters (SC). The occurrence frequencies of the 75 characters range from 30.2 to 1170.43 per million (*Mean * = 326.89, *SD * = 278.11). The total number of strokes in each character (range 6–9 strokes, *Mean* = 6.8, *SD* = 0.92) was counterbalanced among three kinds of characters. Within each structure pairs (left–right characters or top–bottom characters), no radical was repeatedly used. We analyzed occurrence frequency of each radical in the whole corpus. T-Test of radical occurrence frequency between LRC and TBC showed a nonsignificant difference between them [$t (1, 25) = 0.00, p = .999$]. The ANOVA analysis did not show significant difference among three types of structure in frequency [$F (2, 72) = 0.034, p = 0.966$], and in number of strokes [$F (2, 72) = 0.00, p = 1$].

All characters were presented with Song font both in upright and inverted orientations. They were displayed on ViewSonic monitor with the screen resolution set to $1024\,\times \,768$ pixels and a refresh rate of 60 Hz. The presenting position of stimuli on the screen were either 1 deg away (foveal condition) or 4 deg away (parafoveal condition) from the fixation cross. Manipulating eccentricity of faces in visual field may help for dissociating configural from featural processing (McKone 2004). Retinal sizes for characters were therefore set to $1.6\,\times \,1.6 \hbox { deg}^{2 }$ in the fovea and $3.2\,\times \,3.2 \hbox { deg}^{2 }$ in the parafovea. All stimuli were presented in Python.

### Design and Procedure

**Fig. 1:**
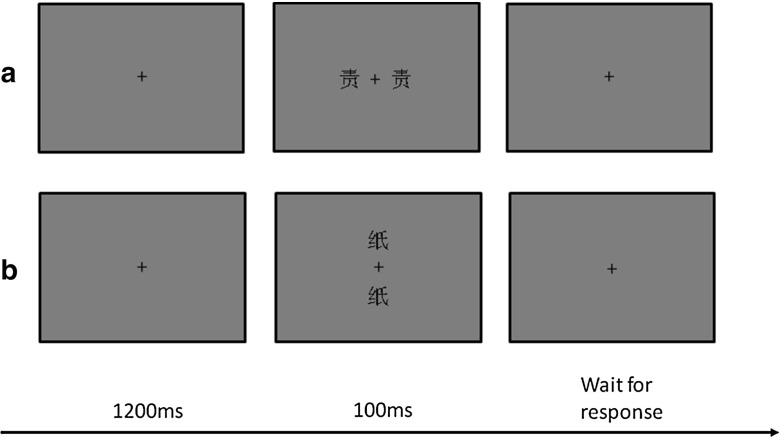
Illustration of two sample trials. **a** Horizontal arranged display. In this example trial, the stimuli are *top–bottom* characters. **b** Vertical arranged display. In the example trial, the stimuli are *left–right* characters. In the experiment, display arrangement was a blocked factor and character structure was a random factor

Participants were tested in a dim and quite room with their heads on a chin rest to maintain the viewing distance at 60 cm. They were instructed to perform a two-alternative same/different task. In the beginning of each trial, a cross was presented at the center of the screen. Participants were asked to maintain fixating at the cross during the whole block. After 1200 ms of the fixation screen, a pair of characters with same structure and orientation was then presented for 100 ms on two sides of the central fixation (see Fig. 1a). Each pair of characters was randomly presented in either foveal or parafoveal vision. Participants were asked to judge whether or not the two characters are the same, pressing the ‘z’ key with the left hand if they were the same and pressing‘/’ key with the right hand if not. An auditory feedback was played to indicate if the response was correct or not. After each block, averaged accuracies and reaction times (RTs) of this block were shown to participants to make sure they were motivated to perform well in the task. The block would have to be retested if the accuracies were lower than 70% or if the average RTs were longer than 900 ms. A total of ten blocks were retested in six participants. Less than three blocks were retested for each participant in the experiment. Conditions in each block were randomized.

The whole experiment included 2400 trials in total and lasted about 90 min for each participant on average. There were two kinds of arrangements for each pair of characters in the experiment: vertical arrangement with one character above the fixation and the other below the fixation, and horizontal arrangement with one character to the left of the fixation and the other to the right of the fixation (see Fig. 1b). Thus, two arrangements were assigned into two sessions so that we could control the interaction of the deployment of spatial attention with the structure of character (i.e. LRC or TBC). The order of two sessions was counterbalanced among participants. Each session included 40 blocks with 30 trials within each block. Character orientation (i.e. upright or inverted) was random across blocks. Within a block, both character structure and eccentric condition were random across trials. There were 100 trials for each condition combination, half of which were ‘same’ trials and the other half were ‘different’ trials. Both sessions were preceded by 4 blocks of practice using a different set of characters from the main tests. Participants have right to choose to finish the two sessions on the same day or on different days. There were 12 participants chose to do the experiment on different days.

**Table 1:** Mean RTs and A’ for upright and inverted characters in the foveal and parafoveal fields

Inversion	LRC		TBC		SC	
	*M*	*SE*	*M*	*SE*	*M*	*SE*
*Foveal field*						
Upright						
RT	449.22	12.62	442.49	12.67	434.42	12.61
$A'$	0.97	0.00	0.97	0.00	0.98	0.00
Inverted						
RT	487.85	12.61	479.66	13.77	465.39	12.18
$A'$	0.94	0.01	0.96	0.00	0.96	0.00
*Parafoveal field*						
Upright						
RT	471.40	12.19	465.30	11.35	453.98	12.19
$A'$	0.96	0.00	0.97	0.00	0.97	0.00
Inverted						
RT	511.81	14.54	500.18	13.45	486.90	12.63
$A'$	0.92	0.01	0.95	0.01	0.95	0.00

### Analysis and Results

Three dependent variables were included into current analysis. First, we analyzed performance among different conditions on mean RTs. The mean RTs from correct trials were computed for each subject. Second, bias-free sensitivity index (*A*’) was calculated with the below equation (1) for each condition instead of accuracy. Previous studies have indicated that *A*’ is a better estimation of performance in a matching task (Hsiao and Cottrell 2009; Wong et al. 2012). For the calculation of *A*’ (see Eq. 1), correct responses to ‘different’ trials were designated as ‘hits’ and incorrect responses to ‘same’ trials were designated as ‘false alarms’. Third, the magnitudes of inversion effects for three structures of characters were calculated by subtracting the RTs of inverted characters from those of the upright ones (i.e. $\Delta$ RT); and the *A*’s of inverted character from those of the upright ones (i.e. $\Delta A$’). For both measurements, higher value means larger size of inversion effect. The magnitudes provide a measure of the relative deterioration across three structures of characters due to inversion. ANOVA was conducted to analyze all three variables (orientation, structure, and eccentricity). Mean RTs and *A*’ for horizontal and vertical arrangement conditions were averaged for statistical analyses (see Table 1).

1 $$\begin{aligned} A'= & {} 0.5 + \left[ {~sign\left( {H - F} \right) \frac{{{{(H + F)}^2} + \mid H - F\mid }}{{4\max \left( {H,F} \right) - 4\textit{HF}}}~} \right] \nonumber \\ H= & {} \hbox {hit rate}, F = \hbox {false alarm rate} \end{aligned}$$

**Fig. 2:**
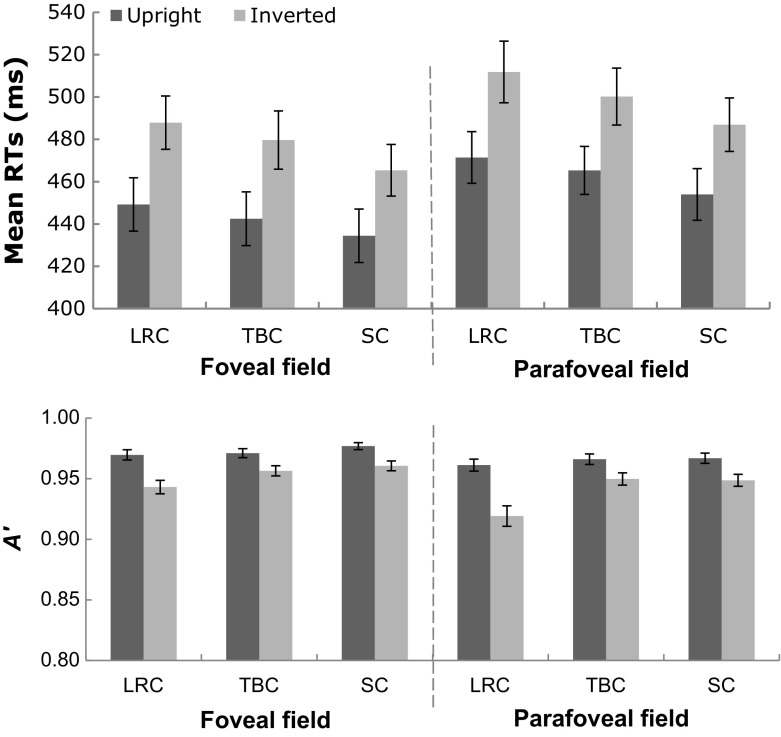
Mean RTs (*top* panel) and A’ (*bottom* panel) for upright and inverted characters in the foveal and parafoveal field. *Error bars* represent standard errors

*Reaction times* To test the impact of inversion on the RTs, a three-way ANOVA was applied on correct RTs with orientation (upright vs. inverted), structure (LRC, TBC, vs. SC), and eccentricity (foveal vs. parafoveal) as within-subject factors. The analysis revealed a main effect of eccentricity ($F (1, 19) = 137.32, p = .000, \upeta _{p}^{2 }= .88$), a main effect of structure ($F (2, 38) {=} 111.88, p {=} .000, \upeta _{p}^{2 }{=}.86$), a main effect of orientation ($F (1, 19) = 155.59, p = .000, \upeta _{p}^{2 }= .89$), and an interaction between structure and orientation ($F (2, 38) = 5.54, p = .015, \upeta _{p}^{2 }= .23$). There are no other significant interactions. Post *hoc* comparisons for the structure by orientation interaction show much longer responses to inverted characters of three structures than upright ones (LRC: $\textit{MD} = 39.52, \textit{SE} =3.57, p = .000; \hbox {TBC: } \textit{MD}= 36.03, \textit{SE} =3.15, p = .000; \hbox {SC: } \textit{MD} =31.95, \textit{SE} =2.71, p = .000$), see Fig. 2.

*Response sensitivity* A three-way ANOVA was applied on *A*’ with orientation (upright or inverted), structure (LRC, TBC, or SC), and eccentricity (foveal or parafoveal) as within-subject factors. There were a main effect of orientation ($F (1, 19) = 57.58, p = .000, \upeta _{p}^{2 }=.74$), a main effect of eccentricity ($F (1, 19) = 22.00, p = .000, \upeta _{p}^{2 }=.54$), and a main effect of structure ($F (2, 38) = 19.06, p = .000, \upeta _{p}^{2 }=.50$). Significant interactions were found between eccentricity and orientation ($F (1, 19) = 4.52, p = .047, \upeta _{p}^{2 }=.19$), between eccentricity and structure ($F (2, 38) = 4.76, p = .018, \upeta _{p}^{2 }=.20$), and between orientation and structure ($F (2, 38) = 14.46, p = .000, \upeta _{p}^{2 }=.43$), which suggest both inversion and eccentricity significantly affected response sensitivity. *Post hoc* comparisons for those 2-way interactions revealed lower sensitivity for all inverted than upright characters ($p\hbox {s} = .000$) either in the parafoveal or foveal visual field. *Post hoc *comparisons reveal lower *A*’ for inverted characters of all structure types than upright ones in the fovea (LRC: *MD* = .027, *SE* =.005, *p* = .000; TBC: *MD*= .015, *SE* =.003, *p* = .000; SC: *MD* =.016, *SE* =.004, *p* = .002) and in the parafovea (LRC: *MD* = .042, *SE* =.007, *p* = .000; TBC: *MD* = .016, *SE* =.004, *p* = .001; SC: *MD* =.018, *SE* =.005, *p* = .001), see Fig. 2.

**Fig. 3:**
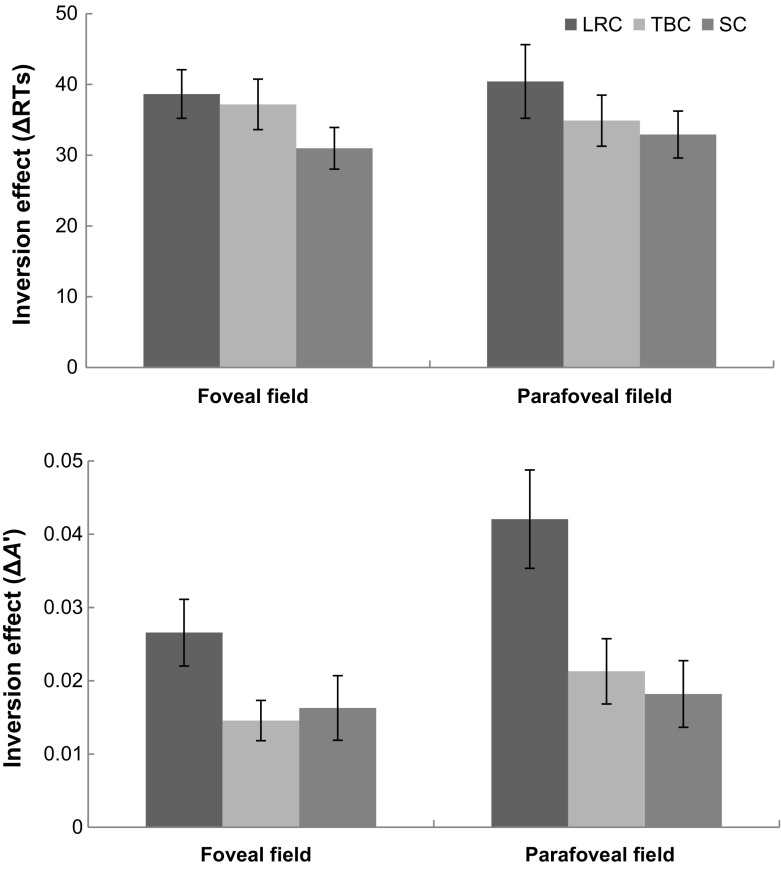
Inversion effects in $\Delta$RTs (*top* panel) and $\Delta$A’ (*bottom* panel) in the foveal and parafoveal field. *Error bars* represent standard errors

*Magnitudes of inversion effect* The magnitudes of inversion effect for characters (refer Table 2) are shown in Fig. 3. The $\Delta \hbox {RTs}$ and $\Delta A$’ are entered into two-way ANOVAs separately with structure (LRC, TBC, or SC) and eccentricity (foveal or parafoveal) as within-subject factors. The ANOVA on $\Delta \hbox {RTs}$ reveal a significant a structure by eccentricity interaction, $F (2, 38) = 5.54, p = .015, \upeta _{p}^{2 }= .23$. Neither a main effect of eccentricity nor a main effect of structure was significant. To detect the structure effect, simple tests for the structure by eccentricity interaction reveal larger magnitude of the inversion effect for LRC than SC (*MD* = 7.67, *SE* = 2.68, *p* = .010) and larger for TBC than SC (*MD* = 6.21, *SE* = 2.82, *p* = .040) in the fovea. These differences cross structures are not observed in the parafovea. There is no eccentricity difference for each type of structure. The ANOVA on $\Delta A$’ showed a significant main effect of eccentricity ($F (1, 19) = 4.52, p = .047, \upeta _{p}^{2 }=.19$) and a main effect of structure ($F (2, 38) = 14.46, p = .000, \upeta _{p}^{2 }=.43$). No structure by eccentricity interaction was found on $\Delta A$’. *Post hoc* comparisons of main effects of structure and eccentricity were conducted. $\Delta A$’ of inversion effect is higher for LRC than TBC (*MD* = .019, *SE* = .004, *p* = .001) and SC (*MD* = .017, *SE* = .005, *p* = .005), but no significant difference in $\Delta A$’ is found between TBC and SC. $\Delta A$’ is overall larger in the parafovea than in the fovea (*MD* = .006, *SE* = .003, *p* = .047).

**Table 2:** Magnitude of inversion effect in reaction times ($\Delta$RTs) and bias-free sensitivity ($\Delta$A’) in the foveal and parafoveal fields

Eccentricity	LRC		TBC		SC	
	*M*	*SE*	*M*	*SE*	*M*	*SE*
Fovea						
$\Delta \hbox {RT}$	38.64	3.44	37.18	3.56	30.97	2.94
$\Delta A$’	0.03	0.00	0.01	0.00	0.02	0.00
Parafovea						
$\Delta$RT	40.41	5.20	34.88	3.61	32.92	3.32
$\Delta A$’	0.04	0.01	0.02	0.00	0.02	0.00

## Discussion

The main purpose of the present study is to determine whether the magnitude of the character inversion effect is dependent on what we call ‘radical structure’. Specifically we measured the impact of the hierarchical level and the familiarity of radical-organization. We used the inversion effect as a measure of configural processing, and a set of three character structures that allowed us to tease apart the effects of structure level and familiarity. The structure familiarity effect has not been investigated so far in the literatures of character recognition. The present study for the first time examined differences in structure familiarity within characters. Our findings showed robust inversion effects for all three types of characters, which are consistent with the findings by Wang et al. (2011). The magnitude of the inversion effect varied with character radical structure. In detail, the inversion effect in RTs was larger to compound (left–right and top–bottom structure) than simple characters, and the response sensitivity was higher to left–right characters relative to top–bottom characters. Taking these findings together, we suggest that configural structure rather than relational configuration is the nature of configural processing of Chinese characters.

Additionally, in the current results, the overall inversion effect, at least on response sensitivity, was higher when the characters were presented in the parafovea compared to that of the fovea condition, which is consistent with McKone’s (2004) finding on face inversion effect. As what one would expect, people generally perform worse at discriminating parafoveal characters. The increment of inversion effect in the parafoveal condition could be simply contributed by more severe impairment in discriminating inverted characters than discriminating upright characters. The processing of inverted characters may rely on only feature processing which could be more dependent on spatial proximity (McKone 2004; Pelli et al. 2004).

Previous findings found that the inversion effect was more profound in the domain that people have gained expertise, or in other words, after they become very familiar with the configuration relations of features of the members (Gauthier and Tarr 1997; Gauthier et al. 1998). By taking advantage of the variation of radical structure of Chinese character, our finding that the inversion effect was stronger for left–right characters is consistent along this perceptual expertise framework, suggesting sensitivity to the configural structure could be modulated through the familiarization of the relation of feature positions even across different subset of objects within a specific domain. Yeh et al. (2003) investigated the effect of learning experience on perceiving Chinese character by comparing various groups with different learning experiences. They found a developmental trend changing from local features to global structures from illiterate to skilled readers. Yeh et al. (2003) explained that learning Chinese characters is a formation process of perceptual units; the size of the units increases with learning experience. This indicated that the local features such as the number of strokes and positions of strokes in radicals become less important to character recognition through learning. The configural structure is determined by the relative positions of radicals rather than strokes (Yeh et al. 2003). The weak inversion effect in simple characters obtained in the present study provides further support for the sensitivity to configural structure. The radical-configural structure is not needed for processing simple characters because a simple character consists of only one radical and has a very simple structure. Its perceptual unit is the radical itself. Inversion may to less degree disrupt configural processing of simple characters. For a skilled reader, perceiving a character by extracting overall configural structure reduces processing load and improves efficiency of character recognition. Taft and his colleagues (Taft et al. 1999; Taft and Zhu 1997) proposed an activation model to illustrate processing of Chinese characters. The orthographic subsystem in the model constitutes four levels: feature, radical, character, and multi-character. When a character is visually presented, low level features (i.e. strokes and stroke combinations) register early in the subsystem. This activates the linked representations at the radical level that then send activations to the character representations. Taft et al. (1999) suggested that the radical representations are position-specific such that the character representations are directly activated via a combination of position-specific radicals from the radical level (i.e. via the combination of the left and right radicals, and top and bottom radicals). Ding et al. (2004) extends the model, specifying the representation levels of simple characters and compound characters. They pointed that radicals and simple characters are represented at the same level; representations of compound characters are activated by position-specific radicals and simple characters.

According to the activation model (Ding et al. 2004; Taft et al. 1999), Chinese characters are recognized via combination of position-specific radials. This is compatible with the explanation of the configural structure. In other words, radicals are indeed organized into different structures that contain radical-positional information. As for the current study, radical processing and structural processing are sufficient for illustration of mechanism underlying recognition of simple characters and compound characters in upright and inverted orientation. Specially, configural structure plays an essential role in recognizing the upright compound characters, which is modulated by structure familiarity (Yeh et al. 2003). The more the structure is familiar to skilled readers, the more character processing relies on configural structure. This is confirmed by the different magnitudes of the inversion effect between left–right and top–bottom characters. Furthermore, the present study considers that radical processing is inevitable in character recognition, as suggested by Zhang et al. (2008) that the configural processing and feature processing are implemented in parallel in Chinese character recognition. The radical representations, as depicted in the extensive activation model by Ding et al. (2004), are activated by simple characters as well. Recognition of inverted simple characters cannot be explained with the configural structure account. Under this circumstance, radical processing contributes recognition of this type of characters. Inversion disrupts normal processing of radical and therefore leads to decrease in processing efficiency.

The structure familiarity we referred in the current study is more about people’s visual experience with character. One has to be cautious in generalizing our findings to other modality, such as, sensorimotor experience. Previous studies suggest that sensorimotor experience could decrease configural processing (Zhou et al. 2012; Tso et al. 2014). For example, artists with extensive face drawing experience attend to facial parts more compared to the control group (Zhou et al. 2012); people with massive writing experience attend to character radicals more compared to those with limited writing experience (Tso et al. 2014).

In the current study, all participants are native-speaking Chinese readers with at least 10 years of writing experience. Thus, the relationship between writing and sensorimotor experience, structure familiarity, and configural processing is beyond the scope of the current study. Nonetheless, this could be an interesting topic for future research that has access to participants with varying sensorimotor experience with characters.

In conclusion, the present study demonstrates through the inversion effect, that character recognition involves extraction of configural structure as well as radical processing rather than multiple levels of relational configurations. Compound characters are recognized via the extraction of configural structure that is modulated by structure familiarity, while recognition of simple characters depends on radical processing.

## References

[CR1] Cai Q, Brysbaert M (2010). SUBTLEX-CH: Chinese word and character frequencies based on film subtitles. PLoS One.

[CR2] Chen YC, Yeh SL (2015). Binding radicals in Chinese character recognition: Evidence from repetition blindness. Journal of Memory and Language.

[CR3] Ding G, Peng D, Taft M (2004). The Nature of the Mental Representation of Radicals in Chinese: A Priming Study. Journal of Experimental Psychology: Learning, Memory and Cognition.

[CR4] Gauthier I, Tarr MJ (1997). Becoming a Greeble expert: Exploring mechanisms for face recognition. Vision Research.

[CR5] Gauthier I, Williams P, Tarr MJ, Tanaka J (1998). Training greeble experts: a framework for studying expert object recognition processes. Vision Research.

[CR6] Guo X-C (1999). Influence of patial frequency, number of strokes, and word frequency to Chinese character recognition. Chinese Journal of Ergonomics.

[CR7] Hsiao J-H, Cottrell GW (2009). Not all visual expertise is holistic, but it may be leftist the case of Chinese character recognition. Psychological Science.

[CR8] Hue C (2003). Number of characters a college student knows. Journal of Chinese Linguistics.

[CR9] Kao C-H, Chen D-Y, Chen C-C (2010). The inversion effect in visual word form processing. Cortex.

[CR10] Lui H-M, Leung M-T, Law S-P, Fung RS-Y (2010). A database for investigating the logographeme as a basic unit of writing Chinese. International Journal of Speech-Language Pathology.

[CR11] Maurer D, Le Grand R, Mondloch CJ (2002). The many faces of configural processing. Trends in cognitive sciences.

[CR12] McKone E (2004). Isolating the special component of face recognition: Peripheral identification and a Mooney face. Journal of Experimental Psychology: Learning, Memory, and Cognition.

[CR13] Pelli DG, Palomares M, Majaj NJ (2004). Crowding is unlike ordinary masking?: Distinguishing feature integration from detection. Journal of Vision.

[CR14] Peterson, M. A., & Rhodes, G. (2003). Analytic and holistic processing–The view through different lenses. In M. A. Peterson, & G. Rhodes (Eds.), *Perception of faces, objects, and scenes: Analytic and holistic processes* (pp. 3–10). Cambridge, New York: Oxford University Press.

[CR15] Sagiv N, Bentin S (2001). Structural encoding of human and schematic faces: Holistic and part-based processes. Journal of Cognitive Neuroscience.

[CR16] Schotter ER, Angele B, Rayner K (2012). Parafoveal processing in reading. Attention, Perception and Psychophysics.

[CR17] Suk-Han Ho C, Wai-Ock Chan D, Lee S-H, Tsang S-M, Luan VH, Ho CS-H (2004). Cognitive profiling and preliminary subtyping in Chinese developmental dyslexia. Cognition.

[CR18] Taft M, Zhu X (1997). Submorphemic processing in reading Chinese. Journal of Experimental Psychology: Learning, Memory and Cognition.

[CR19] Taft M, Zhu XP, Peng DL (1999). Positional specificity of radicals in Chinese character recognition. Journal of memory and Language.

[CR20] Tanaka JW, Sengco JA (1997). Features and their configuration in face recognition. Memory and cognition.

[CR21] Tso RVY, Au TK, Hsiao JH (2014). Perceptual expertise: Can sensorimotor experience change holistic processing and left side bias?. Psychological Science.

[CR22] Vasilev, M.R. & Angele, B. (2016). Parafoveal preview effects from word N + 1 and word N + 2 during reading: A critical review and Bayesian meta-analysis. *Psychonomic Bulletin and Review*. doi:10.3758/s13423-016-1147-x 10.3758/s13423-016-1147-x 27576520

[CR23] Wang M-Y, Kuo B-C, Cheng S-K (2011). Chinese characters elicit face-like N170 inversion effects. Brain and cognition.

[CR24] Wang M, Perfetti CA, Liu Y (2003). Alphabetic readers quickly acquire orthographic structure in learning to read Chinese. Scientific Studies of Reading.

[CR25] Wong AC-N, Bukach CM, Hsiao J, Greenspon E, Ahern E, Duan Y, Lui KFH (2012). Holistic processing as a hallmark of perceptual expertise for nonface categories including Chinese characters. Journal of Vision.

[CR26] Yeh, S.-L. (2000). Structure detection of Chinese characters: Visual search slope as an index of similarity between different-structured characters. *Chinese Journal of Psychology, 42*(2), 191–216.

[CR27] Yeh S-L, Li J-L (2002). Role of structure and component in judgments of visual similarity of Chinese characters. Journal of Experimental Psychology: Human Perception and Performance.

[CR28] Yeh S-L, Li J-L, Chen I-P (1997). The perceptual dimensions underlying the classification of the shapes of Chinese characters. Chinese Journal of Psychology.

[CR29] Yeh S-L, Li J-L, Takeuchi T, Sun V, Liu W-R (2003). The role of learning experience on the perceptual organization of Chinese characters. Visual Cognition.

[CR30] Zhang Y, Qiu J, Huang H, Zhang Q, Bao B (2009). Chinese character recognition in mirror reading: Evidence from eventc—related potential. International Journal of Psychology.

[CR31] Zhang Y, Yuan J, Bao B, Zhang Q (2008). The recognition potential and rotated Chinese characters. Brain Research.

[CR32] Zhou G, Cheng Z, Zhang X, Wong AC-N (2012). Smaller holistic processing of faces associated with face drawing experience. Psychonomic Bulletin and Review.

